# Effect of different types of exercise in adult subjects with fibromyalgia: a systematic review and meta-analysis of randomised clinical trials

**DOI:** 10.1038/s41598-022-14213-x

**Published:** 2022-06-20

**Authors:** Nuno Couto, Diogo Monteiro, Luís Cid, Teresa Bento

**Affiliations:** 1 Sport Sciences School of Rio Maior, Polytechnic of Santarém (ESDRM-IPSantarém), Rio Maior, Portugal; 2 ESECS, Polytechnic of Leiria, Leiria, Portugal; 3 Research Centre in Sports Sciences, Health Sciences and Human Development (CIDESD), Vila Real, Portugal; 4 Life Quality Research Center (CIEQV), Santarém, Portugal

**Keywords:** Diseases, Health care, Health occupations, Rheumatology

## Abstract

Exercise has been recommended for fibromyalgia treatment. However, doubts related to exercise benefits remain unclear. The objective of this study was to summarise, through a systematic review with meta-analysis, the available evidence on the effects of aerobic, resistance and stretching exercise on pain, depression, and quality of life. Search was performed using electronic databases Pubmed and Cochrane Library. Studies with interventions based on aerobic exercise, resistance exercise and stretching exercise published until July 2020 and updated in December 2021, were identified. Randomized controlled trials and meta-analyses involving adults with fibromyalgia were also included. Eighteen studies were selected, including a total of 1184 subjects. The effects were summarised using standardised mean differences (95% confidence intervals) by random effect models. In general, aerobic exercise seems to reduce pain perception, depression and improves quality of life; it also improves mental and physical health-related quality of life. Resistance exercise decreases pain perception and improves quality of life and moreover improves the physical dimension of health-related quality of life. It was also observed that resistance exercise appears to have a non-significant positive effect on depression and the mental dimension of health-related quality of life. Studies revealed that stretching exercise reduces the perception and additionally improves quality of life and health-related quality of life. However, a non-significant effect was observed on depression. We conclude that exercise may be a way to reduce depression, and pain and improve the quality of life in adult subjects with fibromyalgia and should be part of the treatment for this pathology.

## Introduction

Fibromyalgia (FM) is a chronic rheumatic disease of unknown cause characterised by generalised musculoskeletal pain, fatigue, anxiety, and depression^1–5^. These symptoms, among others, result in a reduced quality of quality of life in this population^6^. Depression, in particular, has a high prevalence among this population^7^ and is of great importance on the overall prevalence of psychiatric comorbidity amongst the FM population compared to the general population^8^.

Currently, FM is treated through both pharmacological and non-pharmacological means^9^. Exercise has been widely applied in the clinical field^10^ and is considered a non-pharmacological approach to the treatment of this pathology^3^. In fact, and according to Hagen et al.^11^, there is empirical evidence that exercise reduces the symptoms of most musculoskeletal pathologies, including FM. Indeed, several studies have found that different types of exercise (i.e., aerobic, resistance, and stretching) contribute positively to the quality of life of subjects with FM, enhancing the reduction of pain and depression^12–16^.

However, although systematic reviews and meta-analyses have proliferated in this area, it has been found that the evidence is of low to moderate quality^12^, which led us to question the accuracy and quality of the information provided. Moreover, previous studies failed to report or analyse variables related to the amount of exercise (frequency, volume, or duration)^17^, which may ultimately not provide the most accurate deduction regarding the effects of exercise in FM, and may lead to conclusions such as the ‘low therapeutic validity of exercise in FM’^18^. For these reasons, clarifying the exercise prescription in this population is essential.

Moreover, this information becomes central since adults with FM have a very low rate of adherence to exercise^1^. This occurs not only because of the exacerbation of symptoms, but also because of the contradictory information regarding the exercise these patients receive from the professionals who are part of their treatment team (e.g., rheumatologist, general practitioner, nurse, or physiotherapist)^19^.

In summary, and according to the recommendation of several authors who encourage others to further study this topic^16,20,21^, it was intended to perform a systematic review and meta-analysis to summarise the evidence on the effects of different types of exercise (aerobic exercise—AE, resistance exercise—RE, and stretching exercise—ST) in pain, depression, and quality of life in people with fibromyalgia, in order to contribute to the clarification of the most adequate exercise prescription in this population and to the integration of exercise in the treatment of FM.

## Methods

The present study followed the PRISMA (Preferred Reporting Items for Systematic Reviews and Meta-Analyses) guidelines^22^.

According to what is recommended by the PRISMA protocol^22^, an exploratory search was carried out and we identified PubMed and Cochrane as fundamental for the completion of the study. From this initial search, the descriptors were also defined for the identification of studies.

The search was performed between May and July 2020, and updated in December 2021, using the terms Fibromyalgia (#1) and Exercise (#2) (#1 And #2) in PubMed (213 references) and in the Cochrane Central Register of Controlled Trials (540 references) in all fields, in English, with no restriction on the date of publication.

Potentially relevant articles were searched in the reference lists of the manuscripts obtained in the search, and other systematic reviews and meta-analyses were included if they contained relevant data.

The present study was registered in the PROSPERO database, under the number CRD42020188457.

### Eligibility criteria

The eligibility criteria of the studies were determined according to the PICOS (Population, Intervention, Comparison, Outcomes, and Study Design) strategy, as follows:

#### Population

Subjects aged 18 years old or older, diagnosed with fibromyalgia according to the criteria of American College of Rheumatology^23^.

#### Intervention

Randomised control trials (RCTs) with interventions based on one of the following types of exercise: aerobic (AE), resistance (RE), or stretching (ST) performed out of water. Interventions that included more than one exercise typology were excluded, as well activities performed in hot water, because the use of hot water causes the reduction of pain and stiffness and relaxes the muscles^24^.

#### Comparison

All studies included a comparison of at least one type of exercise (i.e., AE, RE, ST), with another type of exercise, with another form of treatment or with groups of subjects who maintained their daily activities without any type of treatment.

#### Outcomes

All included studies evaluated at least one of the following outcomes: pain; depression, and quality of life, regardless of the instrument used.

#### Type of study

RCTs comparing AE, RE, or ST, with a control group receiving no treatment or usual care, were included.

### Study identification

After the studies were identified, there was an initial screening based on titles and abstracts, followed by selection through reading of the full text of the manuscripts. The search was carried out by two researchers independently. In case of the conflict, another element was included to achieve a final decision on the inclusion or exclusion of RCTs. Subsequently, all studies were read in full to obtain the final selection of studies.

### Data extraction

The following data was extracted from the selected studies: country of origin, authors, design, number of participants, age, gender, type of exercise, intensity, symptoms, adherence, and conclusions of the study.

### Quality of study and risk of bias

The quality of the included studies and the issues related to the risk of bias were evaluated, through the Cochrane Collaboration Risk of Bias Tool^25^.

Two reviewers assessed the quality of the studies, and differences between both reviewers were resolved by mutual agreement or by a third reviewer. The kappa concordance index Cohen^26^ between two reviewers for each of the criteria was determined,

### GRADE assessment

The strength of the evidence was assessed using the Grading of Recommendations, Assessment, Development and Evaluation (GRADE)^27^ system through the GRADEpro. Quality of evidence for meta-analyses began at the high level and was downgraded to lower levels of evidence when risk of bias, inconsistency, indirectness, imprecision or publication bias were present.

The overall certainty of evidence for the effectiveness of exercise interventions for changes in FM symptoms is presented in Tables 1, 2, and 3. Two investigators rated the certainty of each treatment comparison independently and resolved discrepancies by discussions and, if necessary, consulted with a third party

**Table 1:** Summary of findings table of aerobic exercise intervention for fibromyalgia symptoms in adults.

Outcome	Anticipated absolute effects* (95% CI)	No. of participants (studies)	Certainty of the evidence (GRADE)	Information statements
Pain	SMD **1.31 SD lower** (1.79 lower to 0.83 lower)	407 (9 RCTs)	⨁◯◯◯ ^a^^,b,c^ Very low	AE may decrease pain symptoms in adults with FM
Depression	SMD **0.55 SD lower** (0.97 lower to 0.12 lower)	178 (5 RCTs)	⨁◯◯◯ ^a^^,c,d,e^ Very low	AE may decrease depression symptoms in adults with FM
FIQ	SMD **0.37 SD lower** (0.6 lower to 0.14 lower)	302 (4 RCTs)	⨁◯◯◯ ^a^^,c,e^ Very low	AE may decrease the impact of the FM on quality of life
Mental component HRQOL	SMD **1.03 SD higher** (0.46 higher to 1.61 higher)	94 (2 RCTs)	⨁◯◯◯ ^a^^,c,d,e^ Very low	AE increase Mental Component HRQOL symptoms in adults with FM
Physical component HRQOL	SMD **1.03 SD higher** (0.46 higher to 1.61 higher)	94 (2 RCTs)	⨁◯◯◯ ^a^^,c,d,e^ Very low	AE increase Physical Component HRQOL symptoms in adults with FM

Significant values are in bold.

*The risk in the intervention group (and its 95% confidence interval) is based on the assumed risk in the comparison group and the relative effect of the intervention (and its 95% interval confidence).

^a^Large number of studies with high risk of bias.

^b^Heterogeneity present and significant.

^c^Differences in interventions and outcomes measures.

^d^N is under 300.

^e^Asymmetry in the pattern of results.

**Table 2:** Summary of findings table of resistance exercise intervention for fibromyalgia symptoms in adults.

Outcome	Anticipated absolute effects* (95% CI)	No. of participants (studies)	Certainty of the evidence (GRADE)	Information statements
Pain	SMD **1.56 SD lower** (2.27 lower to 0.85 lower)	496 (8 RCTs)	⨁◯◯◯ ^a^^,b,c^ Very low	RE may decrease pain symptoms in adults with FM
Depression	SMD **1.2 SD lower** (2.8 lower to 0.41 higher)	210 (3 RCTs)	⨁◯◯◯ ^a^^,b,c,d^ Very low	RE may decrease depression symptoms in adults with FM
FIQ	SMD **1.48 SD lower** (2.24 lower to 0.73 lower)	271 (5 RCTs)	⨁◯◯◯ ^a,b,c,d,e^ Very low	RE may decrease the impact of the FM on quality of life
Mental component HRQOL	SMD **0.3 SD higher** (0.01 higher to 0.58 higher)	211 (3 RCTs)	⨁◯◯◯ ^a,c,d^ Very low	RE increase Mental Component HRQOL symptoms in adults with FM
Physical component HRQOL	SMD **1.05 SD higher** (0.66 higher to 1.43 higher)	211 (3RCTs)	⨁◯◯◯ ^a,b,c,d^ Very low	RE increase Physical Component HRQOL symptoms in adults with FM

Significant values are in bold.

*The risk in the intervention group (and its 95% confidence interval) is based on the assumed risk in the comparison group and the relative effect of the intervention (and its 95% interval confidence).

^a^Large number of studies with high risk of bias.

^b^Heterogeneity present and significant.

^c^Differences in interventions and outcomes measures.

^d^N is under 300.

^e^Asymmetry in the pattern of results.

**Table 3:** Summary of findings table of stretching exercise intervention for fibromyalgia symptoms in adults.

Oucome	Anticipated absolute effects* (95% CI)	No. of participants (studies)	Certainty of the evidence (GRADE)	Information statements
Pain	SMD **1.05 SD lower** (1.79 lower to 0.3 lower)	225 (5 RCTs)	⨁◯◯◯^a,b,c,d^ Very low	ST may decrease pain symptoms in adults with FM
Depression	SMD **0.85 SD lower** (2.13 lower to 0.43 higher)	119 (2 RCTs)	⨁◯◯◯^a,b,c,d^ Very low	ST may decrease depression symptoms in adults with FM
FIQ	SMD **0.92 SD lower** (1.3 lower to 0.56 lower)	123 (2 RCTs)	⨁◯◯◯^a,c,d^ Very low	ST may decrease the impact of the FM on quality of life
Mental component HRQOL	SMD **0.42 SD higher** (0.06 higher to 0.79 higher)	120 (2 RCTs)	⨁◯◯◯^a,c,d,e^ Very low	ST increase Mental Component HRQOL symptoms in adults with FM
Physical component HRQOL	SMD **1.05 SD higher** (0.66 higher to 1.43 higher)	120 (2 RCTs)	⨁◯◯◯^a,c,d,e^ Very low	ST increase Physical Component HRQOL symptoms in adults with FM

Significant values are in bold.

*The risk in the intervention group (and its 95% confidence interval) is based on the assumed risk in the comparison group and the relative effect of the intervention (and its 95% interval confidence).

^a^Large number of studies with high risk of bias.

^b^Heterogeneity present and significant.

^c^Differences in interventions and outcomes measures.

^d^N is under 300.

^e^Asymmetry in the pattern of results.

### Data synthesis and analysis

Through the meta-analysis, the mean values of pain, depression, and quality of life were compared with the control groups using RevMan software (5.4.1). Heterogeneity was analysed using the statistics of *x*^2^ and *I*^2^, where a value of p < 0.05 or a value of *I*^2^ > 50% indicate considerable heterogeneity^28^. We generated the effect sizes using Hedges’ g score for each trial to assess the magnitude of the treatment effect. To correct for small size bias, we computed the bias-corrected Hedges’ g score for each measure.

The random effects model for an inverse variation was used to calculate the mean difference and the 95% confidence interval.

## Results

### Results of the systematic literature search

**Figure 1:**
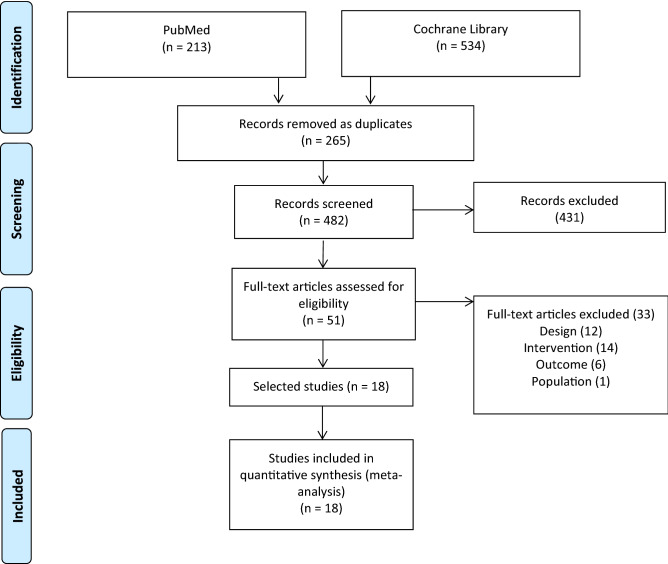
PRISMA flowchart of article inclusion.

The results of the systematic review are summarised in the PRISMA flowchart (Fig. 1). Out of 747 initially screened records, 265 were duplicates, 431 were excluded after title and abstract review. A the full-text review of 33 studies, we were finally left with 18 studies^29–46^ included in the meta-analysis.

### Study characteristics

The total sample of individuals consists of a majority of female subjects (97.46% = 1154) with few male subjects (2.53% = 30), in a total of 1,184 subjects over 18 years old. The selected studies were published between 1996^29^ and 2019^31^. Most studies were of European origin (50% = nine), six were from Brazil (33.33%), and three were from North America (16.66%). Regarding adherence, the studies revealed that there retention was between 77.77%^32^ and 91.66%^33,34^, with an average value of 83.15%.

Ten studies reported the use of RE^30–32,34–40^ and nine reported the use of AE^29,34,36,37,41–45^. Intervention with ST was identified in five studies^30,33,35,38,46^.

The interventions had a duration between three weeks (Hooten) and 24 weeks^45^. Regarding to the frequency, twice a week, was the most frequent, varying between daily frequency^34^ and five times a week^43^.

The duration of the sessions included in the interventions group ranged from 20 minutes^36,41,44^ and 60 minutes^35,37,39,40^.

Taking into account the different types of training, the intervention protocol is quite varied, with indications regarding the structure of the sessions described in Table 4. Regarding the variables under analysis, pain was analysed, through the MPI (Multidimensional Pain Inventory), PCS (Pain Catastrophizing Scale), VAS (Visual Analogic Scale), the pain subscale of the (Fibromyalgia Impact Questionnaire (FIQ), and the Short Form Health Survey (SF-36). Depression was assessed by the Beck Depression Index (BDI) and by the Hospital Anxiety and Depression Scale (HADS). Quality of life was evaluated using the FIQ and SF-36 questionnaire.

**Table 4:** Characteristics of the included studies.

Author/year	Participants	Intervention	Outcomes	Conclusions
Wigers, Stiles, and Vogel 1996^29^	AE: 20 people with FM CG:20 people with FM	*Intervention*: AE Time was gradually increased up to, and decreased down from, four periods of high intensity training at 60–70% of maximum heart rate (altogether 18–20 min). The programme started with a 23 min music session, comprising warming up and two peaks of high intensity training, each of three–four min duration. This was followed by 15 min of aerobic ‘games’ (different types of tag, ball games etc.), representing two high intensity periods of five six min each, with four min of rest in between. The programme ended with warming down and thoroughly stretching out *Comparison*: treatment-as-usual (CG) *Duration*: 45 min three times a week for 14 weeks	Pain (VAS)	Compared to CG, AE induced short-term FM improvement in pain, depression and work capacity, but no obvious group differences in symptom severity were seen in the longer term
Jones et al. 2002^35^	RE: 28 women with FM SE: 28 women with FM	*Intervention*: RE, The RE received a supervised, classroom based, progressive physical training programme with muscle strengthening exercises performed in the standing, sitting, and lying positions, without machine weights, initially with four to five repetitions (reps) and progressing to 12 reps by the end of the study *Intervention*: ST Supervised classes meet for 60 min twice per week for 12 weeks. Class began with a low intensity warmup of marching in place or rhythmic dance for 10 min, gentle stretching for 40 min, and guided imagery and relaxation for the concluding ten min *Duration*: twice a week, for 12 weeks	FIQ; Pain (VAS) Depression (BDI); QOL	The results revealed twice the number of significant improvements in the strengthening group compared to the stretching group. Effect size scores indicated that the magnitude of change was generally greater in the strengthening group than the stretching group
Richards and Scott 2002^41^	AE: 67 people with FM CG: 69 people with FM	*Intervention:* AE An individualized AE programme was used, mostly walking on treadmills and cycling on exercise bicycles. Each individual was encouraged to increase the amount of exercise steadily as tolerated *Comparison:* relaxation therapy (CG) *Duration:* AE: Twice a week (12–50 min) for 12 weeks. CG: 2 days a week (60 min) for 12 weeks	FIQ SF-36	People in the exercise group also had greater reductions in tender point counts and in scores on the FIQ
Valim et al. 2003^33^	AE: 32 women with FM SE: 28 women with FM	*Intervention:* AE The AE group underwent a walking programme monitored with frequency meters and supervised by a physiotherapist three times a week, of 45 min duration, for 20 weeks. The walking speed (training load) was determined by the training heart rate. Training heart rate was defined as the load beat immediately preceding the one in which the anaerobic threshold occurred. Each training session was preceded by a warmup period, where patients were instructed to walk freely and slowly for five to 10 min. After each session, the patients were placed in a circle and made rhythmic movements, to promote cooling off, for five min and ST. The ST programme consisted of three sessions a week of 45 min duration each and included 17 exercises using both muscles and joints in a general way, including face, cervical spine, trunk, and extremities. It lasted for the same 20 weeks. Each maximum position was sustained for 30 s. The exercises were chosen to provide for overall flexibility, without increasing heart rate *Duration:* AE: walking programme three times a week, of 45 min duration, for 20 weeks. SE: programme three sessions a week of 45 min duration 20 weeks	FIQ; SF36; Depression (BDI) Pain (VAS)	Aerobic exercise was superior to stretching in relation to depression, pain, and the emotional aspects and mental health domains of SF-36. Patients in the stretching group showed no improvement in depression, ‘role emotional’ and ‘mental health’
Sencan et al. 2004^42^	AE: 20 people with FM PT: 20 people with FM CG: 20 people with FM	*Intervention*: AE AE were performed three times a week for six weeks and each exercise period lasted for 40 min; the first five min were spent for warm-up, the next 30 min for exercises and the last 5 min were spent cooling down *Comparison*: placebo treatment (CG) *Duration*: aerobic exercises on bicycle ergometer for 40 min, three times a week for six weeks	Pain (VAS) Depression (BDI)	This study shows that aerobic exercise had a better therapeutic effect when compared to the placebo group in terms of pain and depression
Bircan et al. 2008^36^	AE: 13 women with FM RE: 13 women with FM	*Intervention*: AE AE for 20 min and increasing up to 30 min as the patient tolerated. Exercise intensity was adjusted to generate heart rates equivalent to 60–70% of age-adjusted maximum heart rate (220¡ age in years). *Intervention*: resistance exercise (RE) RE the upper and lower limb muscles and trunk muscles, initially with four-five reps and progressing to 12 reps gradually. Free weights and body weight were used for strengthening. Patients began with resistance levels they could do easily, and weight was gradually increased according to the patient’s tolerance. Exercise sessions began with a low intensity warm-up of marching in place and gentle stretching for five min, followed by 30 min of muscle strengthening, and concluded with five min of cool-down and stretching *Duration*: AE (20 min-30 min); RE (40 min)¸three times a week for eight weeks	Depression (HADS) SF-36 Pain (VAS)	AE and SE are similarly effective way to improving symptoms of depression and quality of life in FM
Bressan et al. 2008^46^	SE: 8 women with FM CG: 7 women with FM	*Intervention*: ST The treatment was carried out for eight consecutive weeks and consisted of a 40–45 min weekly session. The participants in G1 underwent a treatment based on static muscular stretching of the triceps surae, isquiotibial, gluteal, paravertebral, latissimocondyloideus, pectoral, trapezius and respiratory muscles. Stretching was performed in dorsal decubitus or sitting. The exercises were performed in a series of five reps, remaining in the same position for 30 secs *Comparison*: physical condition programme (CG) *Duration*: ST was carried out for eight consecutive weeks and consisted of a 40–45 min weekly session	FIQ	Muscle stretching may have had a positive impact on FM, with reductions in morning tiredness and stiffness among the patients evaluated
Günendi et al. 2008^43^	AE: 17 women with FM CG: 15 women with FM	*Interventions*: AE The study group performed submaximal aerobic exercise at 60–80% of maximal heart rate *Comparison*: normal daily activities (CG) *Duration*: AE on treadmill lasting 30 min, five times a week for four weeks. Submaximal AE was performed at 60–80% of maximal heart rate	Pain (VAS) Depression (HADS)	There were statistically significant improvements in the intensity of pain and depression
Panton et al. 2009^32^	RE: 15 women with FM CG: 12 women with FM	*Intervention:* RE All participants performed one set of 8–12 reps twice a week on 10 exercises, using nine resistance machines. Participants began training at approximately 50% of their initial 1-RM measurement and were slowly progressed to approximately 100% of their initial 1-RM by the end of the 16 weeks. Once 12 reps were completed on two consecutive workouts, weights were increased by five to 10 pounds for upper and lower body, respectively *Comparison:* chiropractic treatment (CG) *Duration: 16* weeks of RE consisting of 10 exercises performed twice per week	FIQ	In women with FM, resistance training improves strength, FM impact, and strength domains of functionality
Mannerkorpi et al. 2010^44^	AE: 34 people with FM CG:33 people with FM	*Intervention*: moderate intensity AE The target was to achieve 20 min of moderate-to-high intensity exercise. Exercise intensity was based on the subjective perception of exertion, and patients were instructed as to how to rate exertion on the Borg´s Rating of Perceived Exertion (RPE) scale ranging from six to 20. RPE < 12 is considered to correspond to < 40% of the maximal heart rate, while 12 to 13 (moderate) corresponds to 40 to 60% and 14 to 16 (heavy) to 60 to 85% of the maximal heart rate. The groups started with light exercise for 10 min, ranging from nine (very light) to 11 (fairly light) on the RPE scale, after which they performed two-minute intervals of moderate-to-high intensity exercise, defined as exertion ranging from 13 to 15 on the RPE scale, alternated with two-minute low-intensity exercise, defined as 10 to 11 on the RPE scale. This means that the participants walked at different speeds in small groups, and the leaders alternated between them to provide individual instruction *Comparison*: aerobic exercise low intensity (CG) *Duration*: twice a week (10–20 min), 15 weeks	Pain (FIQ) FIQ	No between-group difference was found for the FIQ Pain and FIQ Total
Sañudo et al. 2010^45^	AE: 22 women with FM CE: 21 women with FM CG: 20 women with FM	*Interventions:* AE Participants performed twice a week of 45 to 60 min duration. Each session included 10 min of warm-up activities (slow walks, easy, movements of progressive intensity); 15 to 20 min of steady-state AE at 60% to 65% of HRmax (calculated as 220-age of participant), including continuous walking with arm movements and jogging; 15 min of interval training at 75% to 80% HRmax (6 exercises for one min and half, resting for one minute between them) that included aerobic dance and jogging; and five to 10 min of cool-down activities (slow walks, easy movements, relaxation training) *Comparison:* normal daily activities (CG) *Duration: twice* a week (45–60 min). CE, twice a week (35–45 min) for 24 weeks	FIQ SF-36 Depression (BDI)	An improvement from baseline in total FIQ and SF-36 score was observed in the exercise groups and was accompanied by decreases in BDI scores relative to controls
Hooten et al. 2012^34^	RE: 36 people with FM AE: 36 people with FM	*Intervention*: RE Study participants completed one set of 10 reps at individually specified weight loads where the initial weight loads for the upper and lower extremities generally ranged from 1–3 kg and 3–5 kg, respectively. All individuals were encouraged to increase weight loads by one kg per week during the course of the three-week study period and AE Therefore, the intensity and duration of AE was not advanced using a standardized protocol; rather, study participants were encouraged to gradually increase the intensity and duration of AE to achieve 70% to 75% of maximal heart rate based on age (220 bpm minus age). Study participants engaged in aerobic exercise up to 10 min daily during week 1 (50 min total week one), up to 15 min during week two (1.25 h total week two), and up to 20 to 30 min daily during week three (90 min to 150 min total week three) *Duration*:10–30 min each day for AE and 25–30 min for RE. Both for three weeks	Pain (MPI)	This study found that strength and aerobic exercise had equivalent effects on reducing pain severity among patients with FM
Kayo et al. 2012^37^	AE: 30 women with FM RE: 30 women with FM CG: 30 women with FM	*Interventions*: AE Walking was performed either outdoors or indoors in a gymnasium, depending on the weather. Each session consisted of a warm-up period, stretching (five-10 min), conditioning stimulus, and a cool down period (five min). Every four weeks, walking duration was increased (25–30 min to 50 min), as well as the intensity of the conditioning stimulus [began at 40–50% and progressed to 60–70% of the heart rate reserve by week and RE group followed an exercise protocol consisting of 11 free active exercises, using free weights and body weight performed in the standing, sitting, and lying positions to improve the muscle strength of the upper and lower limbs and trunk muscles. On average, the exercise load and intensity were increased every two weeks, according to the patient’s tolerance and by following the Borg Scale *Comparison:* conventional treatment (CG) *Duration:* AE and RE practice three days a week (60 min) for 16 weeks	Pain (VAS) FIQ SF-36	Patients in the AE and RE groups reported higher scores (better health status) than controls in almost all SF-36 subscales. RE was as effective in reducing pain regarding all study variables; however, the management of symptoms during the follow-up period was more efficient in the AE group
Gavi et al. 2014^38^	RE: 35 women with FM SE: 31 women with FM	*Intervention:* RE Supervised progressive training in the standing and sitting positions using weight machines. The intensity was moderate, with an overload of 45% of the estimated 1RM. Three sets of 12 reps *Comparison*: ST *Duration*: 45 min twice a week, for 16 weeks	Pain (VAS) FIQ SF-36	ST showed greater and more rapid improvements in pain and strength than flexibility exercises
Larson et al. 2015^39^	RE: 67 women with FM CG: 63 women with FM	*Intervention:* RE The group was initiated at low loads (based in 1-RM), and possibilities for progressions of loads were evaluated every three-four weeks. When the participant was not ready to increase exercise loads, she continued exercising at the same load until she was ready to do so *Comparison:* relaxation therapy (CG) *Duration:* resistance exercise *two* days a week (60 min) for 15 weeks. CG two days a week (25 min) four weeks	FIQ SF-36 Pain (VAS)	Significantly greater improvement was observed in: health status (FIQ total score); pain intensity (VAS); significantly greater improvement were observed in the health related quality of life (SF-36 PCS)
Ericsson et al. 2016^40^	RE: 67 women with FM CG: 63 women with FM	*Intervention:* RE The RE was initiated at 40% of 1 repetition maximum (RM) and progressed up to 80% of 1-RM during the 15 weeks. Possibilities for progression of loads were evaluated every three-four weeks *Comparison:* relaxation therapy (CG) *Duration: twice* a week (60 min) for 15 weeks	Depression (HADS) Pain (PCS)	No significant changes during the study period were found in HADS dimensions (anxiety or depression)
Assumpção et al. 2018^30^	SE: 14 women with FM RE: 16 women with FM CG: 14 women with FM	*Interventions*: ST 12-week supervised exercise programme of 40-min sessions performed twice a week, as suggested by the American College of Sports Medicine and RE *Comparison*: normal daily activities (CG) *Duration*: ST and RE, 12-week 40-min sessions performed twice a week	Pain (VAS) FIQ SF-36	The ST group showed significant improvements in pain, impact on FM symptoms measured by the FIQ total score and quality of life measured by SF-36 physical function, bodily pain, vitality and mental health. After the intervention, the RE group had significant improvements in pain threshold; number of tender points, impact on FM symptoms and quality of life measured by SF-36, as well as better physical function, vitality and mental health compared with baseline
Silva et al. 2018^31^	RE 30 women with FM CG: 30 women with FM	*Interventions*: RE A resistance training programme using weight training for calculating one repetition maximum (1-RM), twice a week for 40 min for a period of 12 weeks. The exercise programme is described: three sets of 12 reps, with an interval of one to two min for recovery; between one set to another, alternating lower limbs. Loads with 60% of 1-RM in the first month, 70% of a new 1-RM test in the second month, and 80% of a new 1-RM test in the third month. Patients were re-evaluated at the end of every four weeks for their load progression *Comparison*: sophrology group (SG) who participated in a relaxation programme based on sophrology *Duration*: RG performed a resistance training programme using weight training twice a week for 40 min for a period of 12 weeks	Pain (VAS) FIQ SF-36	RE led to statistically significant decreases in pain. No differences in pain were found between the groups. RE was more effective than sophrology in improving strength and functional capacity in women with FM

### Quality of studies and risk of bias

The kappa concordance index between the two reviewers was 88.3% for all criteria. All studies presented a low risk due to random sequence generation^29–39,41–46^. Sixteen studies presented a low risk of allocation concealment^29,31–35,37–46^ and two had an unclear risk^30,36^. For the blinding of participants and personnel, 14 studies were characterised as high risk^29,31–42,46^, two as unclear risk and two as low risk of bias^24,37^. For blinding of the outcome assessment, 10 studies were considered low risk^30,32,34,35,38–41,43,44^, six studies as unclear risk^29,33,36,37,42,46^ and two studies as having a high risk of bias^31,45^. For incomplete outcome data, 14 studies had a low risk of bias^29–31,33,34,37,39–46^, three studies had a high risk of bias^35,38,45^ and one had an unclear risk of bias^31^. Finally, all studies were categorised low risk for selective reporting and other bias^29–46^.

**Figure 2:**
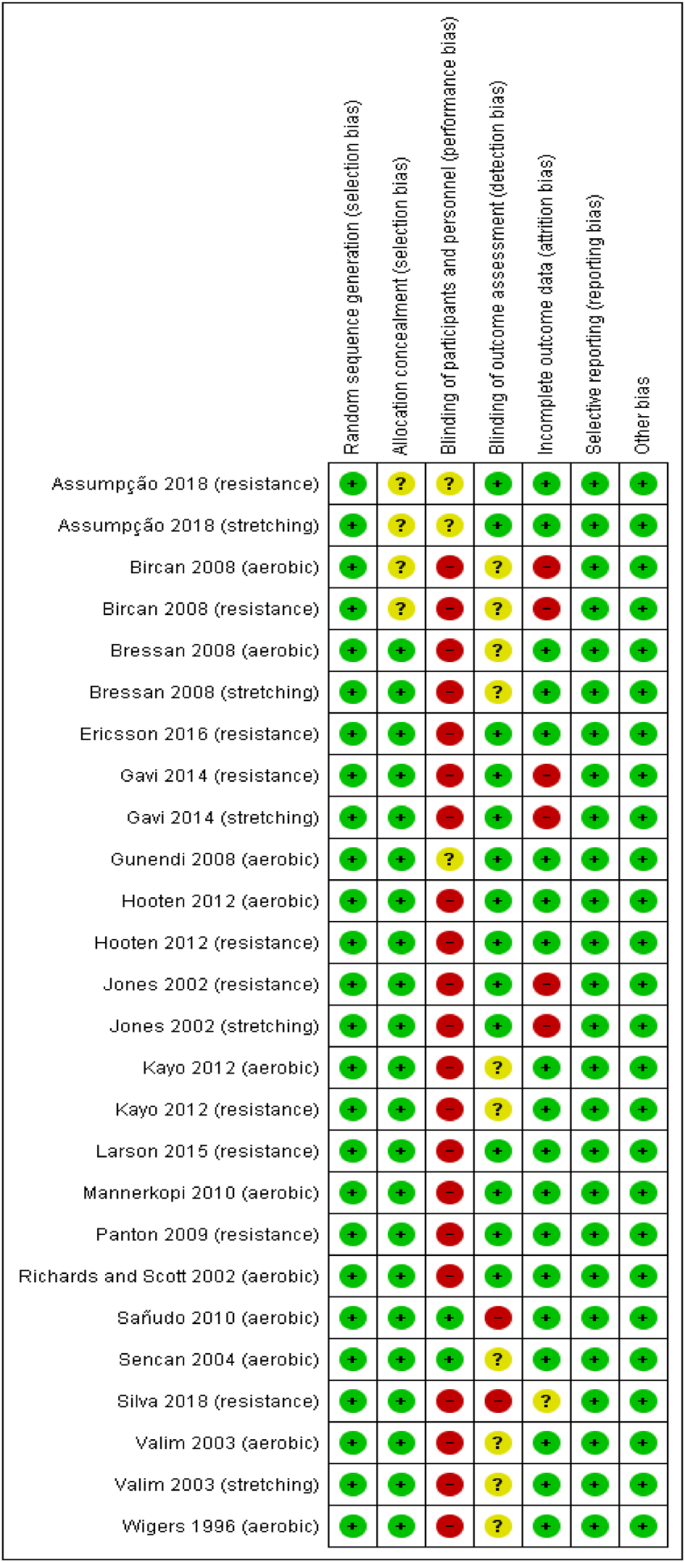
Risk of bias summary: review authors' judgements about each risk of bias item for each included study.

**Figure 3:**
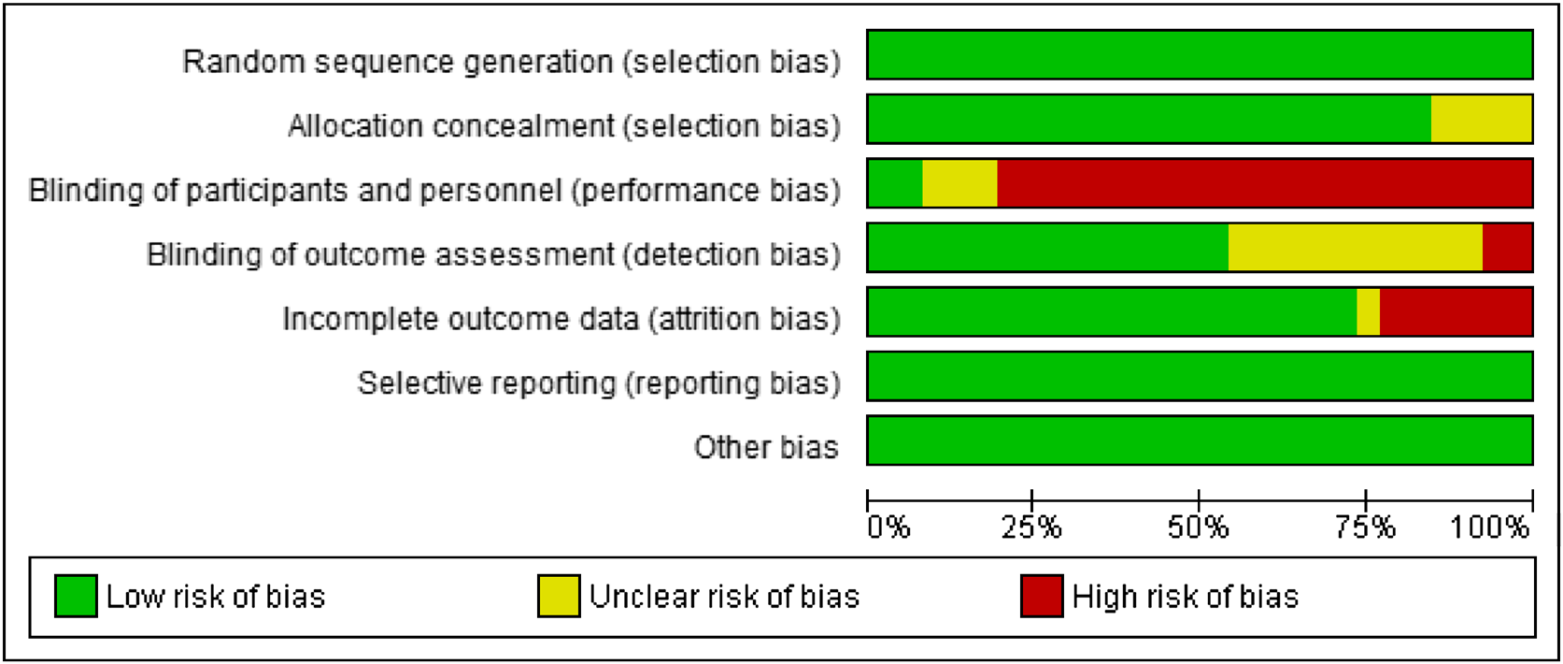
Risk of bias graph: review authors' judgements about each risk of bias item presented as percentages across all included studies.

The individual risk of bias assessment is included in Fig. 2, and the risk of bias assessment for all studies is detailed in Fig. 3.

### Meta-analysis

**Figure 4:**
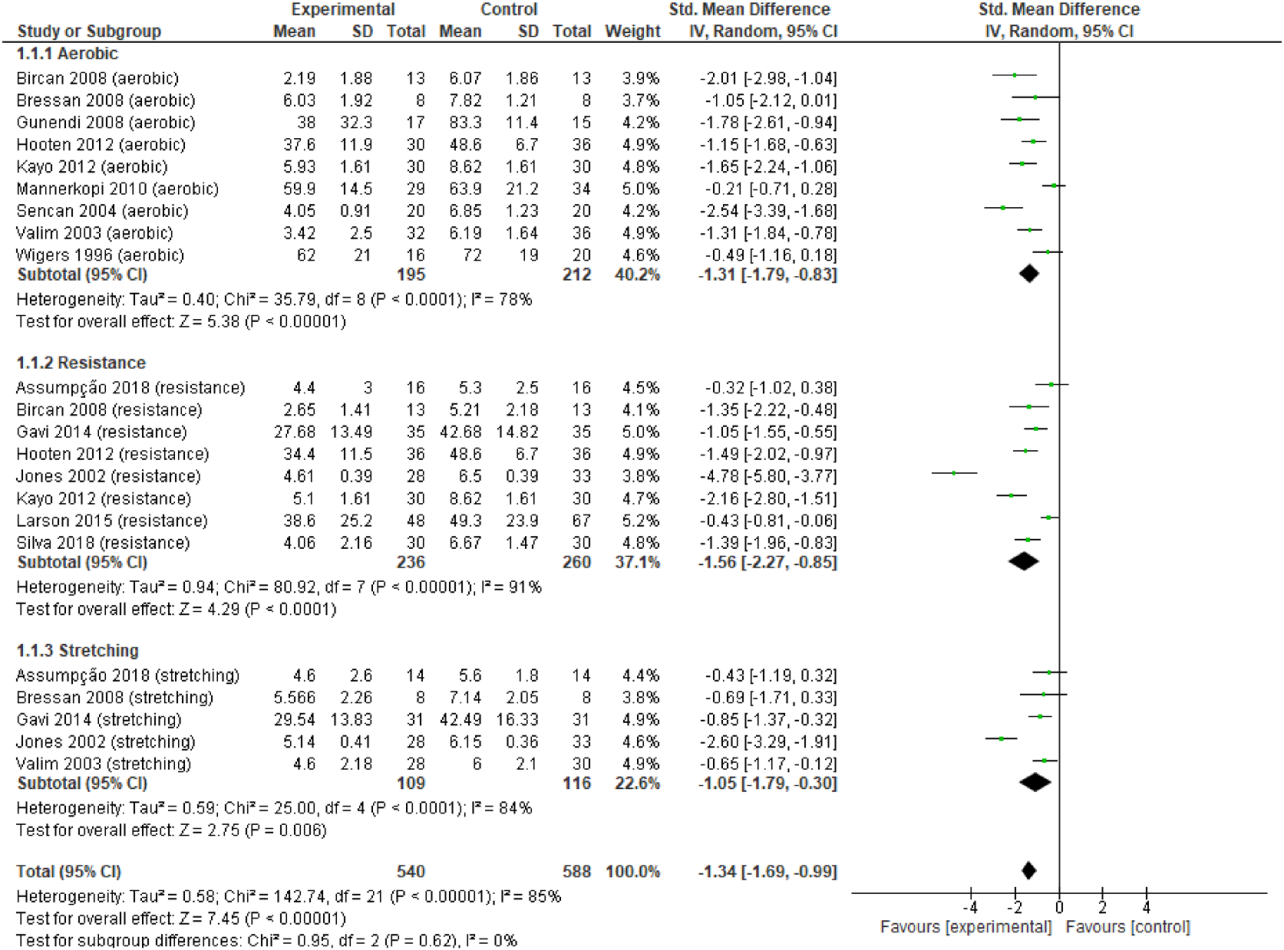
Forest plots showing the effects of training on pain outcomes.

Overall, the results support the conclusion that the three types of exercise have a large and significant effect on pain (Fig. 4) (i.e., > 0.8; overall effect p < 0.05), and each type off exercise did not differ from each other (p > 0.05).

**Figure 5:**
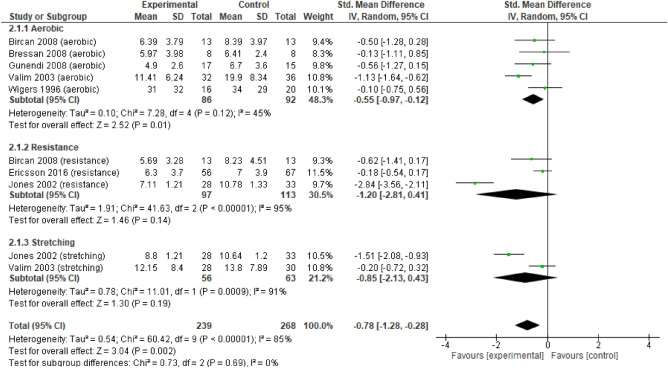
Forest plots showing the effect of training on depression outcomes.

Regarding depression analysis (Fig. 5), no significant differences between the three types of exercise were found (p > 0.05). However, AE was the only one that had a moderate and significant effect in favour of the experimental group; there was a large but non-significant effect for RE and ST.

**Figure 6:**
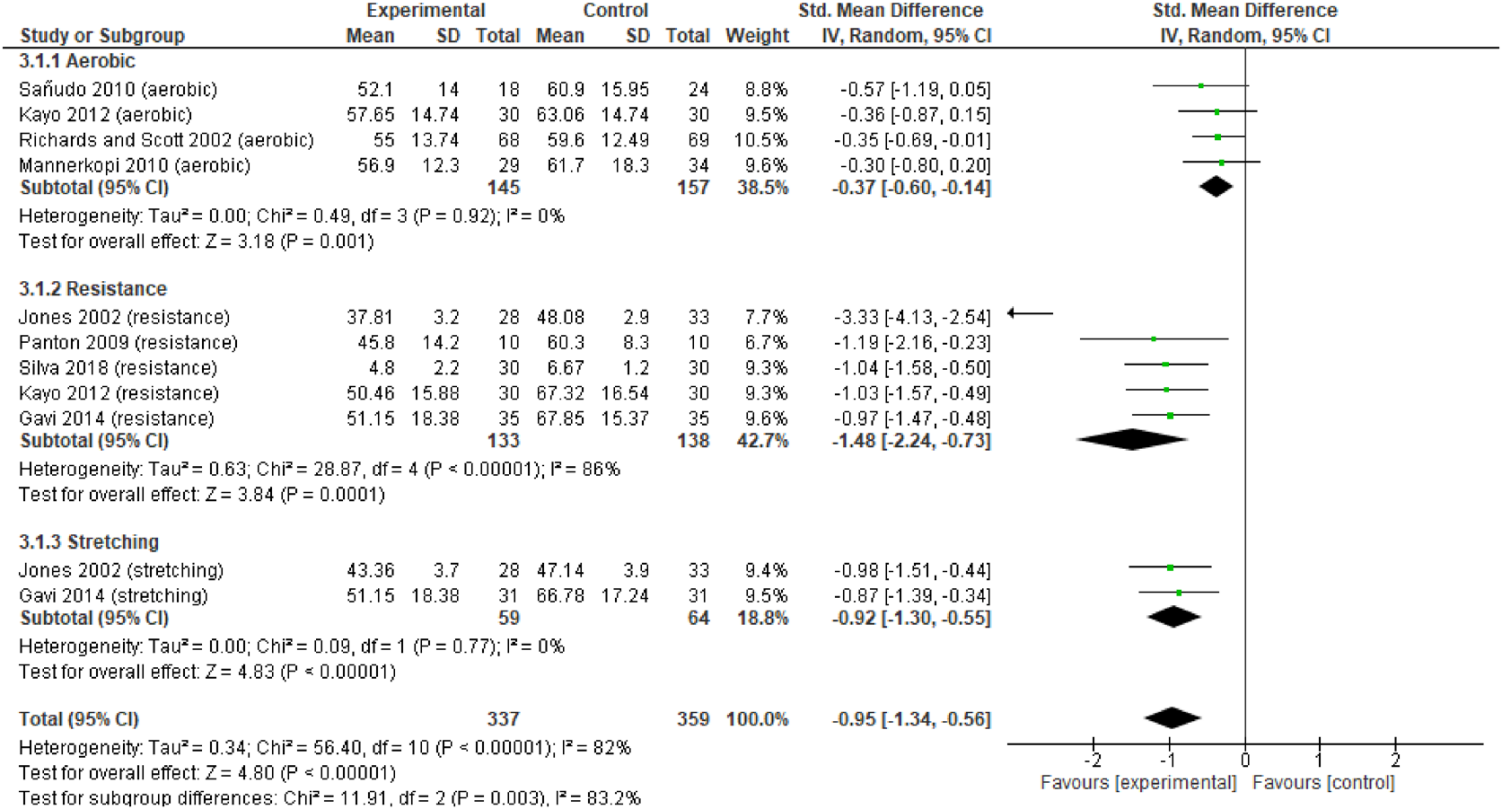
Forest plots showing the effect of training on FM Impact on quality-of-life outcomes.

**Figure 7:**
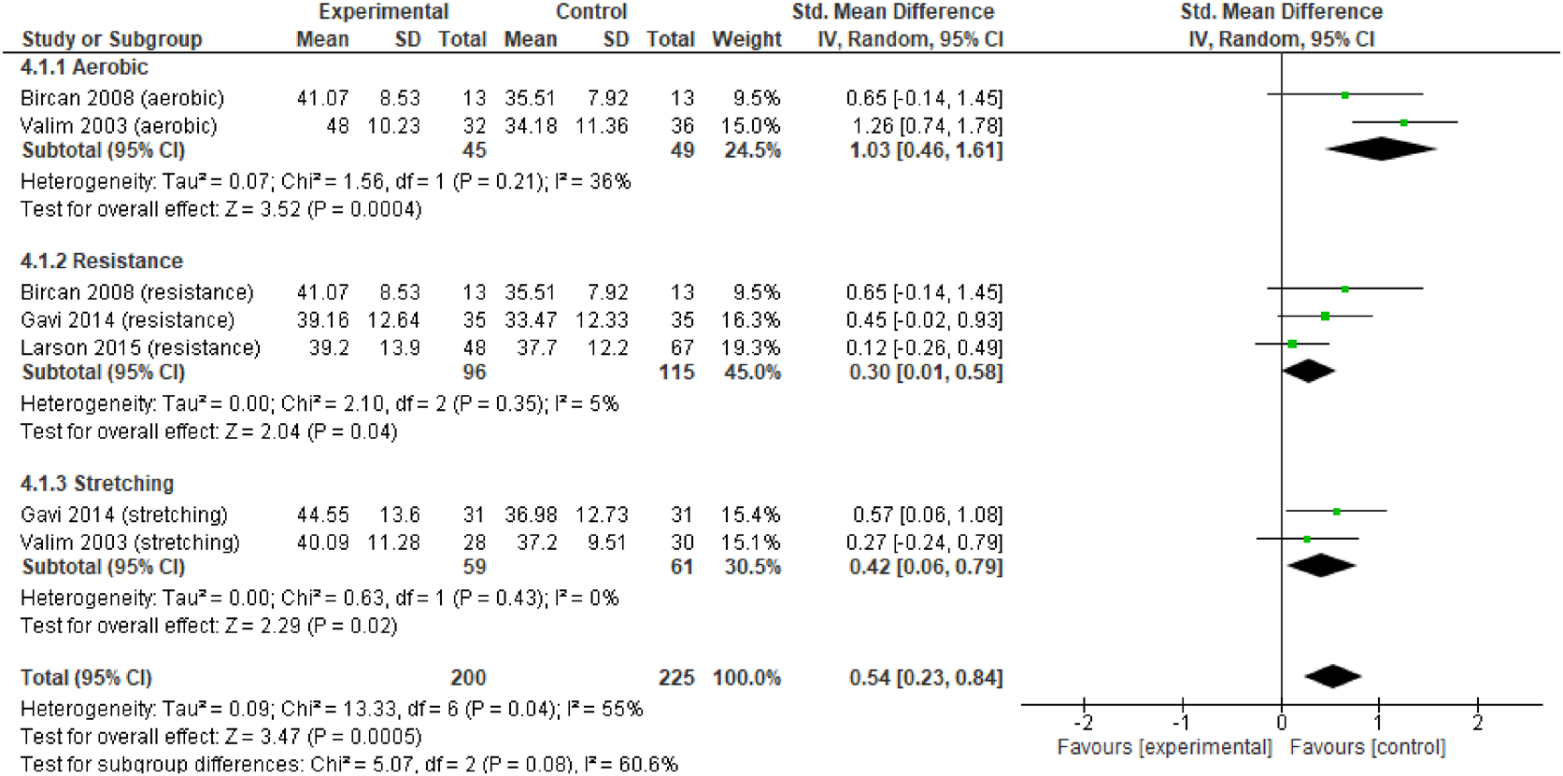
Forest plots showing the effect of training on the Mental Component of HRQOL outcomes.

**Figure 8:**
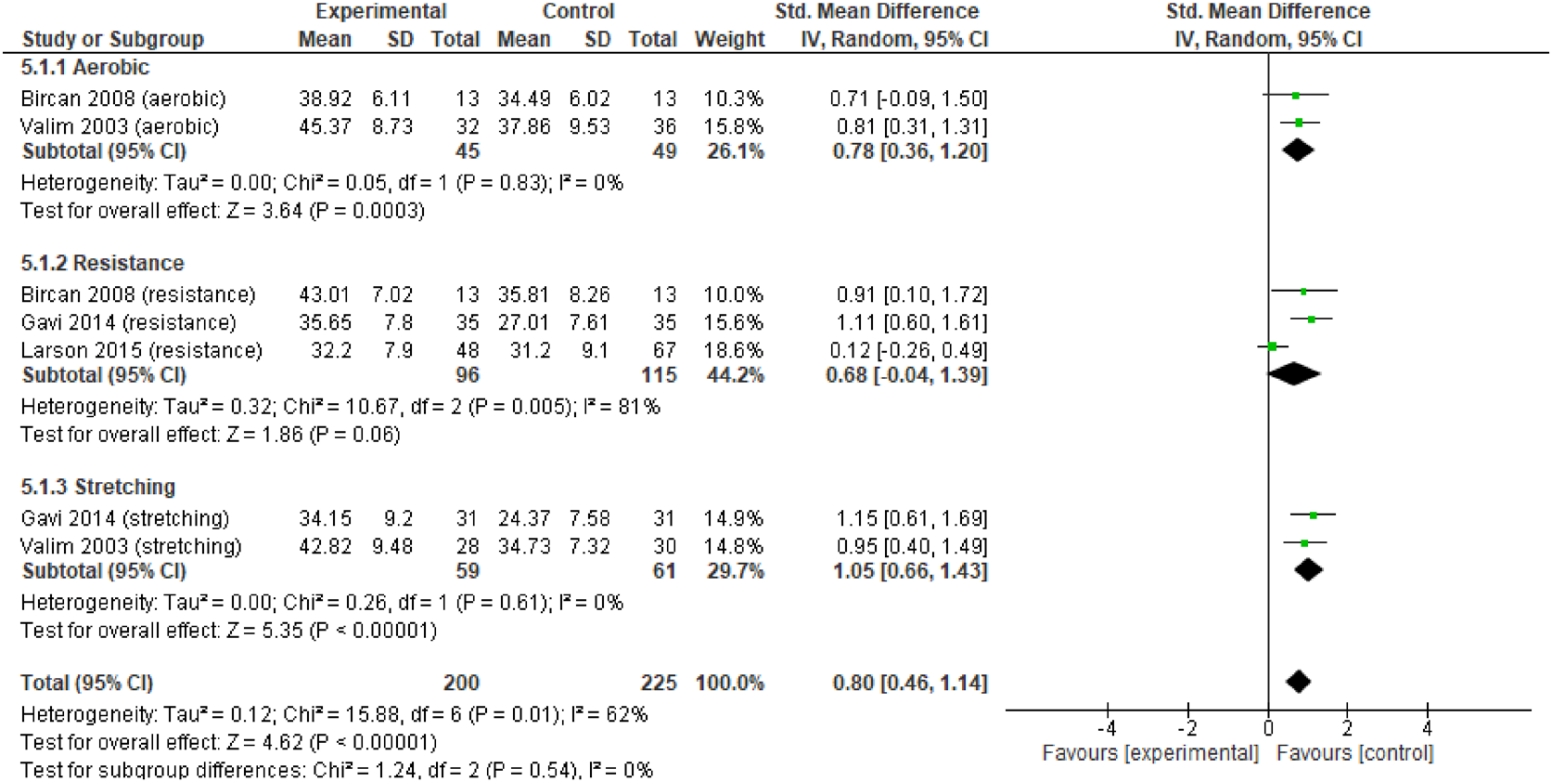
Forest plots showing the effect of training on the Physical Component of HRQOL outcomes.

For quality of life (Fig. 6) there were no differences between the three types of exercise (p > 0.05) in all analysed variables for the FIQ and the Mental and Physical component of SF-36. The according to the FIQ, a large and significant effect of RE and ST was identified. AE had a moderate and significant effect. For the mental component of SF-36 (Fig. 7), AE had a significant large effect, while RE and ST had a moderate significant effect. Regarding the physical component of SF-36 (Fig. 8), there were no significant differences between the three types of exercise. There was a large and significant effect of ST, and a moderate and significant effect of AE. RE had a moderate non-significant effect.

### Heterogeneity

Regarding this point and, according to the Higgins, Thompson, Deeks and Altman^28^ guidelines, it was found through a visual inspection of the pain variable that there was considerable heterogeneity in the studies included in the exercise subgroups of AE, RE, and ST; this was also considerable in the subgroup analysis. For depression, the studies included in the three types of exercise were also shown to be heterogeneous, as well as in the subgroup analysis for this outcome. Regarding FM, both studies involved in the subgroup AE and ST did not prove to be heterogeneous, while the studies involved in the subgroup RE showed considerable heterogeneity; the same occurred in the subgroup analysis. For the health-related quality of life (HRQOL), mental dimension, the identified heterogeneity can be considered not important (*I*^2^ < 0.40) in the three types of exercise as well as the subgroup analysis. For the physical dimension of HRQOL, the studies included in relation to AE and ST were found to be homogeneous, with considerable heterogeneity in the RE subgroup.

## Discussion

The present paper intended to summarise the evidence on the effects of different types of exercise (i.e., AE, RE, and ST) on pain, depression, and quality of life in people with FM.

Regarding AE, the studies indicate a positive influence on the studied variables, as this was the only type of exercise that had a significant effect on them. Indeed, several studies^2,21,47,48^ demonstrated the positive effect of this type of exercise on the major and minor outcomes of FM. For this type of exercise, two perspectives of intervention were observed in the studies: on the one hand, some studies^29,33,35,36,42–44^ recorded the heart rate at intervals of moderate intensity, while in other studies^32,40,41^, subjects were asked to increase exercise intensity to a tolerable threshold, with both having a positive and significant effect on outcomes.

This data allows us to conclude that, even if the exercise starts with a lower intensity than that recommended (e.g., American College of Sports Medicine—ACSM), it is likely to produce benefits in this population. This observation is in line with the study by Häuser et al.^48^, in which the authors state that the quantity and intensity of the AE should be adapted according to the physical condition of each individual, and the disease symptoms of each individual subject should determine the beginning and rate of progression of any exercise^43^. Also, Busch et al.^49^ concluded that it is necessary to initiate AE slightly below an individual’s physical capacity, gradually increasing to moderate levels of intensity, thus avoiding the exacerbation of signs associated with this pathology. In this sense, and according to Bidonde et al.^17^, AE seems to be well tolerated by subjects without increasing pain or other FM symptoms and therefore should be integrated into the treatment programs of FM.

In another perspective, the duration of the intervention could also explain the results. Hauser et al.^48^ states that AE programs in this population must have a minimum duration of four weeks, which in fact is in line with the studies selected in this analysis, as all of them comply with this minimum duration, except for Hooten's^34^ study, which had a duration of three weeks.

Regarding RE, this analysis demonstrated a beneficial effect on all the outcomes analysed, although there was a non-significant positive effect for depression. Busch et al.^2^ also verified the existence of limited evidence of RE on depression. They warned of the promising effect of this type of exercise on this pathology, recommending the need for more and better-quality studies on this type of exercise^48^. Also, in a meta-analysis on RE, Nelson^20^ argues that this type of exercise can be effective and safe in this population, especially when there is a progression from lower intensities. More recently, Vilarino et al.^50^ also verified, in a systematic review of the effects of RE training on the mental health of people with FM, that this type of training potentiated mental health, with special emphasis on reducing depression. However, this study´s inclusion criteria included RCTs where subjects could be sedentary or be part of another type of intervention (i.e., AE; ST). However, despite the limitations on this theme Andrade et al.^47^, recommend that the RE protocol in FM should include a reduced initial intensity (40% of 1-RM) and be gradually increased, with the frequency of exercise between two or three times a week, this aligning, in a way, with the guidelines of the ACSM. This association also argues that the initial intensity should be reduced to the level where the subject does not experience pain. This factor is essential in this population, since, according to Larsson et al.^39^, and through Fleck and Kraemer^51^, the estimation of 1-RM is often performed with perceived submaximal efforts, for reasons of health and safety, which, for this population, due to its symptoms, limits the quantification of this measure.

Despite what has been previously described, the majority of the selected RCTs used the prescription recommended by ACSM for RE in this population. But, like AE, the studies of Assumpção et al.^30^, Jones et al.^35^, Bircan et al.^36^, Kayo et al.^37^, and Larsson et al.^39^ used different methodologies, where pain tolerance and perceived exertion were taken into account in the progression of intensities. However, both perspectives showed a positive evolution of the studied outcomes.

The relation between RE training and depression, as argued by Vilarino et al.^50^ can be influenced by the evaluation methods used in the studies. In fact, although the three RCTs included in this meta-analysis observe a decrease in depression, only the study by Jones et al.^35^ study observed a significant decrease (using the BDI to evaluate depression), while the others (Bircan et al.^36^ and Ericsson et al.^40^) evaluated this outcome using the HADS. For ST, despite the low quality of the information obtained, the studies indicate a positive and statistically significant effect on all outcomes, except for depression. For ST, the ACSM recommends for this population a routine of stretching once or twice a week with progression up to five times a week, for all pain-free muscle groups. Initially, the stretching should be maintained between 10 and 30 s, progressing to the maintenance of each position for up to 60 s. However, in the selected studies, only the study by Assumpção et al.^30^ indicates the ACSM recommendations. This is rare in general, as is the explanation about the progression of intensities and the care in the selection of muscle groups free of pain.

Thus, despite the evident limitations, there seems to be room for the application of ST in subjects with FM, especially due to the beneficial evolution of the studied outcomes. However, in line with Lorena et al.^16^ and Kim et al.^52^, we suggest to further study on this theme, since most of the published papers seem to have low methodological quality and lack exercise standardisation. Although ST appears to be well tolerated, Kim et al.^52^ consider the evidence limited, mainly due to the small number of trials and participants, as well as issues related to a high risk of bias.

Therefore, despite the forms adopted for the progression of intensities, it was found that there were positive and significant effects on the main outcomes, except for depression and the mental dimension of health-related quality of life, which obtained a moderate significant effect.

Consequently, the studies included in the analysis of both symptoms were investigated for these outcomes, verifying that the study by Jones et al.^3^ was the only one in which exercise was performed individually. This point seems fundamental because, according to Busch et al.^53^, subjects in a depressive state with mental health disturbances prefer to practice exercise individually rather than exercise in a group. This can explain the obtained results, and, in our understanding, it should be taken into account in future investigations. Moreover, the symptoms associated with depressive conditions should also be considered since, according to Busch et al.^53^, depression is seen as a significant barrier to exercise.

Still regarding depression, we believe that the difference between observed in the selected studies may also result in the organization of ST sessions. Mainly because the study by Jones et al.^35^, the only one that had a positive and significant effect of ST on depression, the intervention is characterized by 60 min sessions with a 10 min aerobic warm-up (dance) and, on the other hand, in relation to the study by Valim et al.^33^, there is no such indication. This seems to be fundamental since Valim et al.^33^ concludes in his study that the mental component is more influenced by the AE than the ST, as they believe that the AE can induce neurohumoral changes necessary to improve depression, and ST does not. Although we selected the studies for the fundamental part of the intervention, this does not limit the possibility of using other physical capacities in the sessions, especially in the warm-up phase. Thus, considering the results achieved in this outcome and in line with McCain et al.^54^, which attests that ST training should be seen as an intervention because this population benefits from its practice, it is considered fundamental that the study of these variables should be continued in order to effectively clarify the possibility of ST in improving mental health.Through this work, the effect of AE, RE and ST training on possible outcomes was analysed, including a subgroup analysis between the three types of exercise. Although there are already several meta-analyses on this topic, some, for example: Bidonde et al.^13^ and Kelley et al.^3^ studied the effect of exercise on FM without any isolation of the type of exercise, which makes it difficult to identify the real impact that each form of exercise has on the management of this disease.

Thus, to produce a clear analysis, RCT's were included if their main intervention was one of the three types of exercise. Studies were excluded if combined interventions (i.e., several types of exercise) were used or if were carried out in the presence of environmental variables that could influence the effect intervention (e.g., hot water).

Initially, the objective was also to collect evidence about exercise prescription in this population and to determine more effective physical exercise methodologies in the management of this pathology. However, due to the huge diversity of forms of exercise prescription, as well as the presence of samples with different characteristics, combined with the lack of pertinent information in some studies, it became difficult to identify methodologies that would facilitate a greater effect of the different types training on the outcomes analysed.

In addition to the previously identified, we believe the heterogeneity should be addressed. According to several authors, i.e. Higgins and Green^55^ and Bowden et al.^56^, the heterogeneity reflects clinical variability (i.e., variation between participants), methodological variability (i.e., variation between the designs of the studies), variability in sample characteristics (i.e., age, sex, weight), variations in treatment (variability in the thresholds of diagnostic tests) and, additionally, statistical variability (i.e., variation in measures of effect between different studies due to clinical or methodological factors, or both). In this sense, by considering the information collected through the selected references, it can be seen that there is diverse variability in the samples, measures of evaluation of outcomes and exercise prescription, as well as between the types of exercise (i.e., AE, RE, ST), as different forms of prescription within the same exercise group. Thus, taking into account this considerable variability, the statistical effect model was chosen for the statistical analysis. Even with less statistical power than the fixed effects model, it is more realistic for the integration of effect estimates, since it incorporates possible sources of heterogeneity^55,57^.

## Conclusion

Through this systematic review and meta-analysis, was verified that the three types of exercise investigated have a positive effect on pain, depression, and quality of life. The different types of prescription adopted in the analysed studies indicate that exercise is beneficial for this population, especially when considering the principle of individualisation, thereby potentiating a decrease in symptomatology associated with this disease and improving the quality of life in adult subjects with FM. Thus, it is recommended that, when the physical condition of the subjects does not allow the performance of exercise according to the recommendations of generalized international organizations for FM, the physical exercise technicians should adjust the exercise prescription according to individual capacities.

However, the heterogeneity observed in studies´ populations, reported outcomes, and intervention components downgraded the certainty of the evidence, and prevents the drawing of firmer conclusions from the evidence provided. In terms of the future research, it is considered pertinent to continue the investigation into the different forms of exercise in this pathology in larger samples to reduce the bias associated to studies in this population. It is also necessary to understand the adherence behaviour of these subjects in the context of the practice, to create indications for maintaining the practice of this population over time.

In sum, it seems evident that aerobic, resistance, and stretching exercise have a positive effect on pain, depression, and quality of life in adult subjects with FM. We consider that these types of exercise increase health and quality of life in this population and should be considered as part of the treatment for this pathology.

## Supplementary Information

Supplementary Information.

## Data Availability

The datasets used and/or analysed during the current study available from the corresponding author on reasonable request.
